# Biomarkers for Antiplatelet Therapies in Acute Ischemic Stroke: A Clinical Review

**DOI:** 10.3389/fneur.2021.667234

**Published:** 2021-06-10

**Authors:** Adel Alhazzani, Poongothai Venkatachalapathy, Sruthi Padhilahouse, Mohan Sellappan, Murali Munisamy, Mangaiyarkarasi Sekaran, Amit Kumar

**Affiliations:** ^1^Neurology Unit, Medicine Department, College of Medicine, King Saud University, Riyadh, Saudi Arabia; ^2^Department of Pharmacy Practice, Karpagam College of Pharmacy, Coimbatore, India; ^3^Translational Medicine Centre, All India Institute of Medical Sciences, Bhopal, India; ^4^Department of Physiotherapy, Manipal College of Health Professions, Manipal Academy of Higher Education, Manipal, India; ^5^Department of Neurology, All India Institute of Medical Sciences, New Delhi, India

**Keywords:** aspirin, clopidgrel, stroke, prasugrel, ticagrelor, biomarkers, ischemic stroke, resistance

## Abstract

Stroke is one of the world's leading causes of disability and death. Antiplatelet agents are administered to acute ischemic stroke patients as secondary prevention. Clopidogrel involves biotransformation by cytochrome P450 (CYP) enzymes into an active metabolite, and single nucleotide polymorphisms (SNPs) can influence the efficacy of this biotransformation. Despite the therapeutic advantages of aspirin, there is significant inter-individual heterogeneity in response to this antiplatelet drug. In this clinical review, the recent advances in the biomarkers of antiplatelet agents in acute ischemic stroke are discussed. The studies reviewed herein highlight the clinical relevance of antiplatelet resistance, pharmacotherapy of antiplatelet agents predicting drug response, strategies for identifying aspirin resistance, pharmacogenetic variants of antiplatelet agents, miRNAs, and extracellular vesicles (EVs) as biomarkers toward the personalized approach in the management of acute ischemic stroke. The precise pathways contributing to antiplatelet resistance are not very well known but are presumably multi-factorial. It is essential to understand the clinical relevance of clopidogrel and aspirin-related single nucleotide polymorphism (SNPs) as potential predictive and prognostic biomarkers. Prasugrel is a next-generation antiplatelet agent that prevents ADP-platelet activation by binding irreversibly to P2Y12 receptor. There are sporadic reports of prasugrel resistance and polymorphisms in the Platelet endothelial aggregation receptor-1 (PEAR1) that may contribute to a change in the pharmacodynamics response. Ticagrelor, a direct-acting P2Y12-receptor antagonist, is easily absorbed and partly metabolized to major AR-C124910XX metabolite (ARC). Ticagrelor's primary active metabolite, ARC124910XX (ARC), is formed via the most abundant hepatic cytochrome P450 (CYP) enzyme, CYP3A4, and CYP3A5. The integration of specific biomarkers, genotype as well as phenotype-related data in antiplatelet therapy stratification in patients with acute ischemic stroke will be of great clinical significance and could be used as a guiding tool for more effective, personalized therapy.

## Introduction

Acute ischemic stroke (AIS) is an atherosclerotic arterial disease, which is the major cause of death worldwide, leading to an estimated 5.5 million deaths each year (1). The etiology of stroke is established to be multi-factorial. Antiplatelet therapy plays a major role in the primary and secondary prevention of AIS. Most of the stroke occurrence is ischemic and is commonly due to the formation and traveling of the formed emulous into the large vessels, which compromises the blood flow into the brain (2). Neuroimaging is the technique used in the diagnosis and management of the AIS. It plays a major role, as it helps in the differentiation of the hemorrhagic and ischemic stroke where it is important in further management (3). Despite the therapeutic advances in recurrent ischemic stroke management, it affects the quality of life in most people. The treatment failure occurs due to resistance toward antiplatelet therapy or clinically referred to as high on-treatment platelet reactivity (HTPR) (4–6). To overcome this, many platelet function tests are being used, which helps in the platelet function guided antiplatelet therapy, i.e., personalized antiplatelet therapy (7, 8). In recent years, the use of novel biomarkers and pharmacogenetic related data correlating the antiplatelet response and translating it to clinical care has been an area of focus. The incorporation of genomics data along with the clinical markers will be of a paradigm shift in personalized neurology. Hence, this review focuses on interindividual variability and discusses the significance of novel biomarkers and pharmacogenetic data toward the personalized approach in the management of acute ischemic stroke.

## Acute Ischemic Stroke (AIS)

AIS is defined as the occlusion of the brain, retina, or spinal cord supplying arteries, and this results in focal tissue infarction and corresponding sudden neurological deficits. AIS is the leading cause of death worldwide and the third major cause of disability in stroke. More than 7,00,000 cases are estimated to occur worldwide every year (1–3).

For effective diagnosis of AIS, it is important to know about the presence of etiology and risk factors. Most of the patients with etiology have more than two risk factors, and these can be modifiable or non-modifiable. The greater part of the stroke is due to embolisms from heart- cervical arteries or to the atherosclerotic plaque in the aortic arch. The most important mechanism of stroke occurs through intracranial atherosclerosis (2, 9). Based on this mechanism the etiology is subdivided into five major subtypes of (1) large-artery atherosclerosis (embolus or thromboembolism in cervical carotid arteries), (2) cardio embolism (secondary to clot formation in the heart), (3) small-vessel occlusion (lacunar infarct), (4) unusual cause or stroke of other determined causes, and (5) stroke of undetermined causes this classification is based on the Trial of Org 10172 in Acute Stroke Treatment (TOAST), which was developed to categorize the causes of AIS (2, 9). Age is the major factor to which it varies the causes of the presence of stroke in the patients. In children, the occurrence of stroke can be following inflammatory arteriopathy infection. The age of incidence is around 39–49 years and it is higher in men than in women according to the estimate (10). Factors include the following: the presence of hypertension, an increased apolipoprotein B (Apo B) to Apo-A1 ratio, diet, psychological stress, smoking, high alcohol consumption, diabetes, chronic kidney disease, and cardiac conditions like atrial fibrillation (2, 9–13).

The most important thing to note during the diagnosis is the negative factors that mimic the presence of stroke-like migraine, seizures, vestibular disturbance, metabolic disturbance, and also intracranial hemorrhage. Detection based on these symptoms is the first line for the detection of AIS (14). Globally, it is meant that computerized tomography (CT) and rapid access through magnetic resonance imaging (MRI) are the major diagnosing method used for AIS. In Table 1, the diagnostic parameters based on stroke etiology are mentioned (15, 16).

**Table 1:** Etiology and parameters in diagnosis of AIS.

**Etiology**	**Diagnostic parameter**
Cardiac embolism	Echocardiography
	Holter/loop recorder
Atherosclerosis	CT angiography
	MR angiography
	Carotid Doppler ultrasonography
Small vessel disease	Brain MRI
Arterial dissection	CT angiography
	MR angiography
Cerebral vasculitis	CT angiography
	Magnetic resonance angiography
	Catheter angiography
	Cerebrospinal fluid examination
	Brain and leptomeningeal biopsy

### Pharmacotherapy of Antiplatelet Agents Predicting Drug Response

Platelet reactivity phenomena involve platelet adhesion, aggregation, and activation. Various antiplatelet agents like aspirin, clopidogrel, glycoprotein IIb/IIIa antagonists, and P2Y12 agents have been studied to prevent any events of atherothrombosis. However, variability in platelet reactivity and response between subjects is of major concern in antiplatelet therapy. It can result from a variety of factors. Elevated levels of immature platelet count and reactivity affect the response to antiplatelet agents. Drug-based factors include drug–drug interactions (DDIs), dosing, etc. Patient-related factors include compliance, metabolism, comorbidities like diabetes mellitus, obesity, abnormal lipid profile, and smoking habits. The Euro Heart Survey on Diabetes and the Heart (17) revealed patients with coronary artery disease and diabetes possess a higher risk of cardiovascular events and mortality, which explains the altered response to antiplatelet therapy (18); the concurrent occurrence of both diabetes mellitus and chronic kidney disease (CKD) increases the risk even more, creating a demand for highly effective antiplatelet treatment (18, 19). The Platelet Inhibition and Patient Outcomes (PLATO) trial comparing clopidogrel vs. ticagrelor in acute coronary syndrome (ACS) patients has revealed the possibility of harm from H2 receptor blockers with clopidogrel therapy (20). Further comparison studies have supported the use of H2 receptor blockers in the place of Proton Pump Inhibitors (PPIs) to provide GI protection, as the latter is associated with adverse health outcomes (21, 22). Moreover, recurrent strokes are instigated by homocysteine levels, where patients with higher levels show lower response to antiplatelet therapy (23–25) supported by several studies demonstrating the link between hyperhomocysteinemia and platelet activation and insufficient platelet inhibition (26). The recurrent stroke and cardiovascular events can be predicted by baseline homocysteine levels of dual antiplatelet therapy or aspirin alone in the female patients with acute minor stroke or high-risk Transient ischemic attack (TIA) (27). The CHANCE trial (Clopidogrel in High-Risk Patients with Acute Nondisabling Cerebrovascular Events) demonstrated the superior benefits of dual therapy with clopidogrel and aspirin in managing recurrent stroke in patients with high-risk TIA than aspirin alone (28). Thus, in order to prevent atherothrombotic events in patients with high risk, varied antiplatelet mechanisms offered by dual antiplatelet therapy will be of huge benefit (29).

### Aspirin

Several factors alter platelet reactivity and turnover and thus leading to aspirin response variability and “High on-treatment platelet reactivity” (HTPR). Hyperresponsiveness to aspirin is multifactorial with altered pathways. Ageing, type 2 diabetes mellitus (DM), and drug interactions [most common with non-steroidal anti-inflammatory agents (NSAIDs)] at binding site Ser529 of COX-1 reduce the response to aspirin (30) and proton pump inhibitors (PPIs), and myeloproliferative conditions are some of the contributing factors for variability in aspirin responses (31, 32). A variety of platelet-activating mechanisms, elevated levels of platelet production, insufficient COX-1 inhibition, augmented recovery of COX-2 with increased platelet turnover, and elevated levels of aspirin-insensitive agonists may affect the aspirin response at the cellular level. Along with these factors, genetic polymorphisms also play a vital role in altered response to aspirin between patients (33). Reduced response to aspirin is expected after coronary artery bypass graft (CABG) procedure over a brief time affecting the prevention of failure of the thrombotic graft. In such cases, aspirin dosing multiple times per day was found to control the TXB2 generation efficiently in an early study trial (34), which was confirmed by a meta-analysis including 7 Randomised Clinical Trials (RCTs), where therapy with aspirin twice daily has better antiplatelet efficacy in comparison with a daily dose of one per day (35).

### Clopidogrel

This is a prodrug rendering its pharmacological action once metabolized to its active form by Cytochrome 450 and Paraoxonase-1 (PON-1). It is a two-step mechanism. The first step involves the action of CYP2C19, CYP1A2, and CYP2B6 (36). The second step involves CYP3A4, CYP2C9, and the Paraoxonase (PON-1) enzyme. Despite this, dual antiplatelet therapy is efficient in Major Adverse Cardiovascular Events (MACE) prevention and is considered as the norm in clinical management. There occurs substantial levels of recurrent events (~10%) (37). In secondary prevention of cardio and cerebrovascular events, clopidogrel is considered a highly effective antiplatelet therapy, where along with aspirin it acts as the backbone to preventing major adverse cardiovascular events (MACE) (38). However, 25% of patients exhibit only a sub-optimal response to this drug (39). The pharmacodynamics response to clopidogrel exhibit a wide inter-individual variability (40). High platelet reactivity with clopidogrel in patients with DM leads to the impaired antiplatelet response, which is explained by the altered drug pharmacokinetics (41). CYP2C19^*^2 or ^*^3 and PON-1 polymorphisms considerably diminished the platelet response to clopidogrel while the former elevates the risk of MACE in Coronary Heart Disease (CHD) patients after PCI (42). In a meta-analysis conducted with 28 studies across 17 countries in Asia, ABCB1 C3435T polymorphism considerably reduced platelet activity in patients receiving clopidogrel, thereby elevating the risk of bleeding events (43). A recent systematic review and meta-analysis study has recommended genotype testing of ABCB1 C3435T SNP for ACS/CAD patients undertaking PCI to optimize clopidogrel treatment (44). A meta-analysis study has demonstrated the risk of high PR and MACE in patients with vascular risk factors receiving clopidogrel therapy. This substantiates the need for a future individualized method of antiplatelet treatment based on the personal vascular risk factors (45).

### Ticagrelor and Prasugrel

The Platelet Inhibition and Patient Outcomes (PLATO) trial demonstrated ticagrelor given at a maintenance dose of 90 mg bid reduced cardiovascular events in comparison with clopidogrel in ACS patients (20). The POPular AGE trial, involving patients in the ACS, ticagrelor, and prasugrel groups, showed just a 53% adherence rate during the 1-year follow-up, and this was in most part due to the side effects and recognized risk of bleeding events (46). The effect of ticagrelor on health outcomes in diabetes mellitus patient's intervention trial studied ticagrelor versus placebo in addition to aspirin in stable CAD patients with type 2 diabetes, a considerable 15% reduction in ischaemic events was observed with added ticagrelor (47). The ticagrelor 60 mg bid was studied to attain the same pharmacokinetic and pharmacodynamic effect as such of high dose as 90 mg bid in the prevention of cardiovascular events in patients with prior heart attack using ticagrelor compared to placebo on a background of aspirin–thrombolysis in myocardial infarction study (48). A long-term randomized clinical trial comparing standard antiplatelet therapy and individualized antiplatelet regimen based on the pharmacogenetic profile of acute ischemic minor stroke (AIMS) and transient ischemic stroke (TIA) patients in a Chinese population was undertaken to establish evidence to support the importance of genomic profiling to select P2Y12 receptor antagonists in such patients (49).

## Antiplatelet Resistance

Antiplatelet therapy is crucial to the secondary prevention of acute ischemic stroke to prevent Recurrent Ischemic Stroke (RIS) attacks (4). Despite its effectiveness and the proper intake of drugs, to some extent, aspirin or clopidogrel fail to produce pharmacological action, i.e., when it fails to inhibit platelet aggregation due to a reduction in platelet sensitivity and thus leads to recurrent adverse vascular events and this phenomenon led in coining the term “Resistance,” which is now clinically referred as “High on Treatment Platelet Reactivity (HTPR)”: the treatment failure of antiplatelet therapy (4–6, 50). Low or non-responders to antiplatelet treatment are more prone to resistance and are prone to increased risk of suffering RIS events and early neurological deterioration (6, 51, 52).

**Figure 1:**
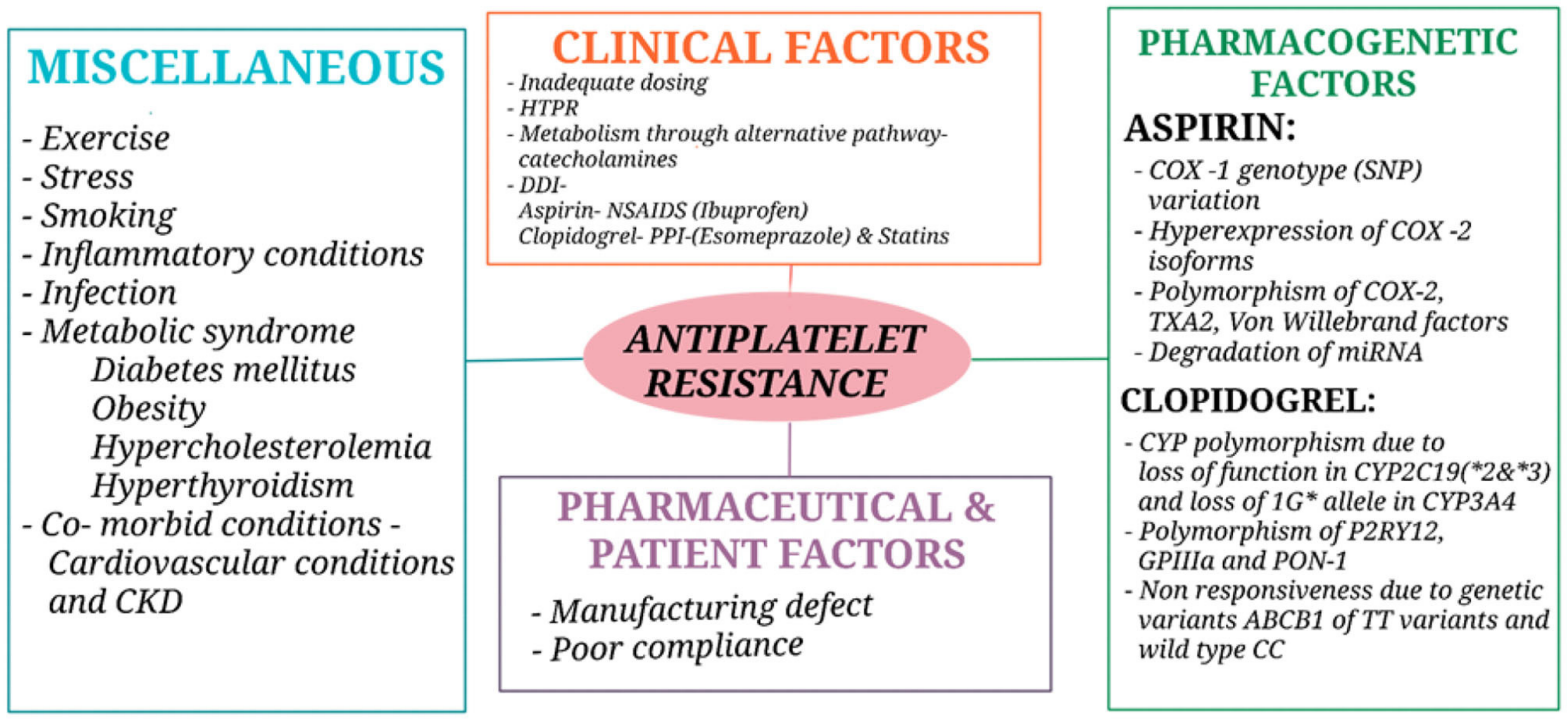
Causes of antiplatelet resistance. CKD, Chronic kidney disease; HTPR, High on-treatment platelet reactivity; DDI, Drug drug interaction; NSAIDS, Non-steroidal anti-inflammatory drugs; PPI, Proton pump inhibitors; COX, Cyclooxygenase; TXA2, Thromboxane A2; miRNA, micro Ribonucleic acid; CYP, Cytochrome; GP, Glycoprotein; PON-1, Paraoxonase 1.

The different approaches used in defining antiplatelet resistance are (1) laboratory resistance—an increase in the levels of thromboxane A2 (TXA2) metabolites due to the inadequate inhibition of TXA2 and platelet aggregation despite antiplatelet therapy (53–55)—and (2) clinical resistance—when there is antiplatelet treatment failure (i.e., a failure to prevent antithrombotic event occurrence in stroke patients) (6, 53, 54). The most important factors for antiplatelet resistance in patients with AIS are due to poor adherence and concurrent use of other cyclooxygenase- 1 (COX- 1) inhibitors (56) and genetic factors like single nucleotide polymorphism (SNP) of the receptors (*P*2*Y*_12_, *P*2*Y*_1_, *GPIIb*−*IIIa*, collagen receptor, TXA2, etc.) and enzymes (COX-1&2). Other causes for resistance include the pharmaceutical preparation, anion efflux pump, interaction of platelets with other cells like endothelial cells or monocytes, accelerated platelet turnover, and activation of an alternate pathway for metabolism (57). Metabolic syndromes like diabetes mellitus because of hyper glycation of platelet protein but prediabetes is independent of resistance (56, 58, 59) hypercholesterolemia, increased body weight (obesity) (60, 61) smoking (62), and interaction with some drugs like Proton Pump Inhibitors (PPIs), e.g., esomeprazole and clopidogrel, and Non-Steroidal Anti-Inflammatory Drugs (NSAIDS), e.g., Ibuprofen and Aspirin (50, 53–55, 57, 63, 64). Examples of antiplatelet resistance causes are shown in Figure 1.

A study on 69 patients on the prognostic value of high platelet reactivity in ischemic stroke depending on etiology based on large- and small-vessel disease concluded that large vessel disease worsens early prognosis and in small vessel disease worsens late prognosis and clinical and functional condition of the patients, thus resistances is also dependent on the etiology of the stroke condition (65). This was confirmed in a 3-year follow-up period study where they also concluded that there is the large-vessel etiology of AIS is associated with the occurrence of adverse vascular events in HTPR patients and it is also associated with large infarct volume in the patients (66) and HTPR also leads in the formation of ischemic lesions in the brain (67). A Cytokine Registry in Stroke Patients (CRISP) study conducted in India based on the response of clopidogrel resistance in ischemic stroke patients has linked female sex and proton pump inhibitors use rather than cytochrome polymorphism (68). In the Chinese population, it was found that clopidogrel resistance due to a polymorphism of the CYP2C19^*^2 allele with or without hypertension and a P2Y12 receptor variant (68, 69) is associated with recurrent ischemic stroke, adverse vascular events, and poor recovery from neurological deficits (70). Another study postulated that CYP2C19^*^2 allele polymorphism or loss of function of CYP2C19^*^3 are at high risk for clopidogrel resistance (71), and thus it can be assumed that the clopidogrel resistance is mostly due to CYP2C19 polymorphism which was conformed in systematic review and meta-analysis by Alakbarzade et al. (71). Therefore, the cause for resistance from antiplatelet therapy is multifactorial, and genetic polymorphisms play a major role in resistance etiology.

Platelet function guided antiplatelet therapy is getting more important because of increased resistance from antiplatelet drugs like aspirin and clopidogrel which is included in most AIS patients, and they experience different adverse vascular events due to the treatment failure. It also helps in the tailored or personalized antiplatelet therapy in the patients who have high on-treatment platelet reactivity and in the early detection of adverse vascular events (7, 8). So, it is important to measure the inhibition of the platelet function in patients with AIS who have HTPR (72). The different platelet function testing methods are bleeding time, light transmission platelet aggregation (LTA), impedance platelet aggregation, lumi-aggregometry, and tests based on platelet function methods combined with viscoelastic tests, such as Thromboelastographs (TEGs)/platelet mapping systems, Rotational Thromboelastometry (ROTEM) platelets, and others, where Flow Cytometry is used to test the platelet activation, and Radio- or Enzyme-Linked Immuno Assay measure the thromboxane A2 metabolites (8, 57, 73–75).

Despite the development of these many types of analyses to test the responsiveness of the antiplatelet therapy there remain several drawbacks, which ultimately create an upcoming challenge. The challenges faced during the Platelet Function Test (PFT)-guided antiplatelet therapy are due to the lack of consistency and standardization, automation, difficulty in the process, and inability to fulfill all the parameter needed in one test; it is also a promising challenge for researchers in making the assays into the clinical laboratory since most do not make through it (76, 77). The accuracy to capture the *in vivo* platelet function with *in vitro* platelet function test assays is still challenging (77). The other parameters reveal equipment that is expensive and time consuming to use in which a high volume of the sample is needed, and all the tests need well-trained staff to run the procedure. It is important to select the relevant test for the particular drug; it must be defined clearly. A study comparing PFT in AIS with antiplatelet therapy concluded that LTA-AA and TEG-AA showed a good correlation for monitoring the aspirin effect. PFA-EPI may be more likely to report resistance. TEG-ADP may not be appropriate for assessing platelet function in clopidogrel users. CYP2C19 genotyping will be the better option for the detection of platelet function (78). Nevertheless, different studies showed different results: a systematic review and meta-analysis of 1,136 participants included two retrospective studies based on platelet function analysis (PFA)-guided antiplatelet therapy in recurrent stroke with or without antiplatelet therapy modified (ATM) actions (79–81). Although there are many challenges, the PFT plays a vital role in the personalized antiplatelet therapy and the prediction of early occurrence of bleeding and adverse vascular events in AIS patients.

### Strategies for Identifying Aspirin Resistance

AR is a multifactorial pathological condition that has many different causes. The aspirin resistance can be identified both clinically and through laboratory methods. Clinically, it can be identified from the occurrence of atherothrombotic events in a patient who is under the therapeutic effect of one dose of aspirin. But this method is limited because it is mostly non-specific and can only be identified retrospectively because the events occur only after the start of the treatment (82, 83). The laboratory monitoring of PFT is based on the platelet aggregation and presence of platelet reactivity which is mentioned above. These PFTs are the most used methods for the detection of aspirin resistance. Despite its limitations, PFT is most specific and considerable over time (84). Aspirin resistance can be relevant with the prediction of concentration of proteinuria in patients with AIS, and these are on aspirin therapy. Thus, proteinuria can be considered as a tool in identifying aspirin resistance (11), and AR is useful as a prognostic marker for cardiovascular disorders and other comorbidities of AIS (85).

## Pharmacogenetic Variants of Antiplatelet Agents

### Pharmacogenetics of Aspirin

Multiple factors contribute to lowered aspirin efficacy (86) with genetic determinants attribute to 30% of cases (87). The patients with C765G (rs20417) polymorphism of COX-2 was established to have lowered risk of adverse cardiovascular events in aspirin users (Odds Ratio (OR): 0.78, 95% CI: 0.70– 0.87) (88). The PlA1/A2 SNP of the GPIIIa receptor gene was studied to be associated with lowered aspirin response. The SNP rs5918 in the ITGB3 gene was significantly associated with an amplified platelet response to aspirin (89).

### Pharmacogenetics of Clopidogrel

Clopidogrel is a widely prescribed drug for the prevention of recurrent ischemic events in patients with ACS or MI due to its efficacy and cost-effectiveness compared to other antiplatelet agents. It is most commonly used along with aspirin as dual antiplatelet therapy in the prevention of atherothrombotic events. However, wide variability occurs between patients in response to clopidogrel therapy, and some even present with clopidogrel resistance. The CYP2C19 polymorphisms are the most common and well-studied polymorphisms associated with clopidogrel response (90). In trial to assess improvement in therapeutic outcomes by optimizing platelet inhibition with Prasugrel–Thrombolysis in myocardial infarction 38 trial, ACS PCI patients with ATP Binding Cassette Subfamily B Member 1 (ABCB1) T-allele homozygotes had adverse cardiovascular events like recurrent stroke and MI (91). Numerous Loss-of-Function (LOF) variants in CYP2C19 affect antiplatelet response to clopidogrel. SNP rs4244285 of CYP2C19^*^2 (92)and SNP rs12248560 of CYP2C19^*^17 contribute to altered clopidogrel response (86). Although, earlier studies have established the minimal association between polymorphisms such as CYP1A2^*^1F and CYP2C9^*^2/3 and response to clopidogrel. The later studies have failed to replicate any significant association (86, 93). Through the pharmacogenomics of anti-platelet intervention (PAPI) study involving 566 subjects, the missense polymorphism (G143E, rs71647871) was demonstrated to affect clopidogrel drug response and reactivity (94). Patients with Paraoxonase 1 192Q-allele homozygotes had reduced clopidogrel response and lowered bleeding complications (HR = 0.4, 95% CI: 0.2–0.8, *P* = 0.006) (88). ABCB1 C3435T variant in PCI patients with homozygous T allele showed significantly lower levels of the drug and hence the antiplatelet activity (95). Recognizing the impact this has on drug metabolism, the clinical pharmacogenetics implementation consortium (CPIC) guideline recommends alternate antiplatelet treatment for ACS/PCI patients estimated to be altered metabolizers of the drug (90).

### Pharmacogenetics of Prasugrel and Ticagrelor

Numerous studies have investigated the association of CYP450 variants in response to prasugrel. SNPs rs4244285 and rs12248560 of CYP2C19 were found to be significantly associated with a prasugrel response. However, no association was established in CYP2C9, CYP2B6, CYP3A4, or CYP1A2 variants related to prasugrel response (96). Ticagrelor is a next-generation P2Y12 inhibitor. It gets disintegrated to an equally effective primary active metabolite, AR-C124910XX via CYP3A4/5 metabolism (97, 98). A genome-wide association study was conducted to detect SNPs associated with Ticagrelor levels and response from the PLATO clinical trial (99). SNP rs56324128 in CYP3A4, rs62471956 SNP in CYP3A43, rs61361928 SNP in UGT2B7, and rs4149056 SNP in SLCO1B1 were significantly associated with decreased levels of ticagrelor plasma concentrations. SNP rs113681054 of the SLCO1B1 gene, CYP3A4^*^1, and CYP3A4^*^22 variants of CYP3A4 were significantly associated with increased plasma ticagrelor concentrations. SNP rs4661012 in Platelet Endothelial Aggregation Receptor-1 (PEAR1) gene was associated with decreased ticagrelor response and SNPs-rs12566888 & rs12041331 in PEAR1 gene was associated with increased ticagrelor response. Where, CYP3A4^*^1, CYP3A4^*^22 variants are related to high inhibition of platelet aggregation (100–102). In Table 2, the association between a pharmacogenetic variant and a drug phenotype is summarized.

**Table 2:** Pharmacogenetic variant association of antiplatelet drugs.

**GENE**	**Ref SNP (rs) number**	**Association**	**Condition**	**Population**	**References**
CYP3A4	rs56324128	Genotype CC is associated with reduced levels of ticagrelor compared to genotype CT.	ACS	European	(101)
SLCO1B1	rs113681054	Allele C in comparison with allele T is associated with elevated ticagrelor levels.	ACS	European	(101)
	rs4149056	Allele T compared to allele C is associated with reduced levels of ticagrelor.	ACS	European	(101)
CYP3A43	rs62471956	Allele G is associated with reduced levels of ticagrelor as compared to allele A.	ACS	European	(101)
UGT2B7	rs61361928	Genotype TT is associated with reduced levels of ticagrelor as compared to genotype CT.	ACS	European	(101)
PEAR1	rs12566888	Genotype TT is associated with elevated response to ticagrelor as compared to genotype GT.	Healthy individuals	Chinese	(102)
	rs4661012	Genotypes GT + TT is associated with reduced response to ticagrelor as compared to genotype GG.	Healthy individuals	Chinese	(102)
	rs12041331	Genotype AA is associated with augmented response to ticagrelor as compared to genotypes AG + GG.	Healthy individuals	Chinese	(102)
	rs12041331	Genotype AA is associated with increased response to ticagrelor as compared to genotype GG.	Healthy individuals	Chinese	(102)
P2RY1	rs1065776	Patients with genotype CT may have elevated risk of aspirin-resistant phenotype as compared to patients with genotype TT.	CAD	European	(103)
		Patients with genotype CT may have reduction in AA-induced platelet aggregation after aspirin treatment as compared to patients with genotype CC.	Healthy individuals	Chinese	(104)
ITGB3	rs5918	Patients with genotype TT may have aspirin-depressed thrombin generation and prolonged bleeding time after aspirin treatment as compared to patients with genotypes CC + CT.	CAD	Poland	(105)
		Patients with genotypes CC + CT may possess elevated risk of lack of aspirin response as compared to patients with genotype TT.	CAD	Poland	(106)
		Patients with genotype TT may have elevated risk of inadequate inhibition of platelet activity as compared to patients with genotypes CC + CT.	CAD	Tunisian	(107)
		Patients with genotype CT may have reduced aspirin mediated platelet inhibition as compared to patients with genotype TT.	CAD	United States	(89)
LPA	rs3798220	Patients with genotype CT may have reduced risk of Myocardial Infarction on aspirin treatment.	Healthy individuals	European	(108)
TBXA2R	rs4523	Patients with genotype AA may have elevated risk of residual platelet reactivity with aspirin treatment as compared to patients with genotypes AG + GG.	Off-pump coronary artery bypass grafting	Chinese	(109)
GP6	rs1613662	Patients with genotype AG may have elevated risk of non-response to aspirin as compared to patients with genotype GG.	CAD	Finland	(110)
GP1BA	rs6065	Patients with genotypes CT + TT may have elevated response to aspirin in men as compared to patients with genotype CC.	Healthy individuals	Japan	(111)
CYP2C19	rs4244285	Patients with allele A may possess an elevated risk of platelet reactivity as compared to patients with genotype GG.	ACS	France	(112)
		Patients with allele A may have increased platelet reactivity index (PRI) vasodilator-stimulated phosphoprotein (VASP) at 1 month of prasugrel treatment as compared to patients with genotype GG.	ACS	France	(112)
	rs12248560	Patients with allele T may have reduced platelet reactivity index (PRI) vasodilator-stimulated phosphoprotein (VASP) at 1 month of prasugrel treatment as compared to patients with genotype CC.	ACS	France	(112)
		Patients with allele T may have a reduced rate of high on-treatment platelet reactivity (HTPR) at 1 month of prasugrel treatment as compared to patients with genotype CC.	ACS	France	(112)
		Patients with allele T may possess escalated rate of hyper-response at 1 month of prasugrel treatment as compared to patients with genotype CC.	ACS	France	(112)
PEAR1	rs41273215	Patients with genotype TT may have reduced levels of inhibition of ADP-induced platelet aggregation compared to patients with genotypes CC + CT.	Healthy individuals	Chinese	(113)
	rs3737224	Patients with genotype TT may have reduced levels of inhibition of ADP-induced platelet aggregation compared to patients with genotypes CC + CT.	Healthy individuals	Chinese	(113)
	rs77235035	Patients with genotype AA may have reduced levels of inhibition of ADP-induced platelet aggregation as compared to patients with genotypes AC + CC.	Healthy individuals	Chinese	(113)
	rs822442	Patients with genotype AA are associated with reduced levels of inhibition of ADP-induced platelet aggregation as compared to patients with genotypes AC + CC.	Healthy individuals	Chinese	(113)
	rs822441	Patients with genotype CC are associated with reduced levels of inhibition of ADP-induced platelet aggregation as compared to patients with genotypes CG + GG.	Healthy individuals	Chinese	(113)
	rs12407843	Patients with genotype AA are associated with reduced inhibition of ADP-induced platelet aggregation as compared to patients with genotypes AG + GG.	Healthy individuals	Chinese	(113)

*CYP3A4, Cytochrome P450 Family 3 Subfamily A Member 4; SLCO1B1, Solute Carrier Organic Anion Transporter Family Member 1B1; CYP3A43, Cytochrome P450 Family 3 Subfamily A Member 43; UGT2B7, UDP Glucuronosyltransferase Family 2 Member B7; P2RY1, Purinergic Receptor P2Y1; ITGB3, Integrin Subunit Beta 3; LPA, Lipoprotein(A); TBXA2R, Thromboxane A2 Receptor; GP6, Glycoprotein VI Platelet; GP1BA, Glycoprotein Ib Platelet Subunit Alpha; CYP2C19, Cytochrome P450 Family 2 Subfamily C Member 19; PEAR1, Platelet Endothelial Aggregation Receptor 1*.

## Biomarkers in Acute Ischemic Stroke

Numerous types of biomarkers are investigated in stroke, including physical, imaging, histological, genetic, electrophysiological, neuronal, and serum markers. Among these, genetic biomarkers can aid in personalizing stroke management through the detection of genetic variations including heritable cerebrovascular disorders. The Trial of Org 10172 in Acute Stroke Treatment (TOAST) classification based on clinical parameters is the currently used method of ischemic stroke classification (114–116). Stroke occurrence is multifactorial with various mechanisms involved in its different subtypes. The development of specific novel and reliable biomarkers will be of great clinical significance. Platelets play a vital role in hemostasis. The human genome is estimated to encode around 1000 miRNAs. More than 100 of these are detected in human sera of healthy individuals and are termed circulating miRNAs (117). miRNAs, endogenous non-coding RNA molecules, are found to be abundant in platelets and are studied to be associated with platelet activity, inhibition, and responsiveness, making them good candidates as biomarkers. They inhibit mRNA translation and are released from platelets upon activation. Several studies have proposed the use of miRNAs as potential biomarkers to study platelet response in patients receiving antiplatelet treatment throughout the course of therapy as it plays a vital role in pathophysiological processes of stroke-related injuries. miRNAs and their target genes are involved in a variety of ischemic stroke pathophysiologies, including angiogenesis and neurogenesis (118). miRNAs are found to target many proteins in various regulatory cell signaling loci and signaling pathways in platelets. Several miRNAs play roles in both intrinsic and extrinsic apoptosis pathways. In the extrinsic apoptosis pathway, miR-21 and miR-25 are found to regulate TNF-α signaling affecting the stroke outcome. Upregulation of miR-155 reduces inflammation via miR-155–CARHSP1–TNF-α signaling (119). As a result, miRNA profiling appears to be a promising diagnostic marker for ischemic stroke in the future. miR-223, let-7c, and miR19a are the most copious platelet miRNAs. Reduced levels of miRNAs like miR-191, miR-126, miR-150, and miR-223 were detected in the plasma of healthy subjects treated with increasing dose of aspirin with prasugrel, indicating miRNAs response to platelet inhibition (120). Similarly, in healthy individuals treated with clopidogrel and ticagrelor, reduced levels of miR-223^*^ and miR-197 were observed (121). The miR-96, miR-107, miR-200b, miR-223 and miR- 495 are significantly associated with platelet activation, secretion, and reactivity (1). miR-128b, miR-124, and miR-1246 have been studied to be associated with ischemic stroke and are detected to be up-regulated in stroke patients compared to healthy subjects (122, 123). In ischemic stroke patients with infarcts >2 cm^3^, the elevated levels of miRNAs like miR-9-5p, miR-9-3p, miR-124-3p, and miR-128-3p were detected through next-generation sequencing technology indicating release of miRNAs with injury (114).

In patients of T2DM with ischemic stroke, the platelet miR-144 level was found to be elevated, while levels of platelet miR-223 and miR-146a were reduced (124). Significant reductions in levels of plasma miRNAs- miR-223, miR-126, and miR-150 were observed in patients treated with more potent antiplatelet agents such as P2Y12 inhibitors (125). Jager et al. (126) in a study on miRNAs- miR-223, miR-150, miR-126, and miR-21 established to be related to platelet function, suggested that these miRNAs may not be used as platelet activation related biomarkers after cessation of P2Y12 inhibitors treatment. Tiedt et al. (127) in their comprehensive study, identified three circulating miRNAs, 125a-5p, 125b-5p, and 143-3p, as potential biomarkers after acute ischemic stroke. Neutrophil extracellular traps (NETs) were detected in plasma and thrombus of ischemic stroke, suggestive a new prognostic biomarker in acute ischemic stroke patients (128, 129).

Numerous evidence from past studies has established the relationship between mean platelet volume (MPV) and cerebrovascular events (130, 131). Some suggested the use of mean platelet volume (MPV) as a potential diagnostic and prognostic biomarker of acute ischemic stroke (132). In certain studies, MPV was detected to be raised both in acute ischemic stroke and certain hemorrhagic strokes (133). The range of MPV and MPV/Platelet count (PC) ratio was studied to be significantly represented in stroke patients than healthy individuals (134, 135). Also the MPV and MPV/PC ratio tests are cost-effective, relatively simple, and can aid risk identification of stroke (136). Along with that, MPV levels are suggested to vary among stroke subtypes depending on the severity of injury and size of the infarct. The levels of MPV and MPV/PC ratio were studied to be significantly higher in atrial fibrillation (AF) stroke than large artery atherosclerosis (LAA) stroke, where both are subtypes of ischemic stroke (137). Hence, it can act as a biomarker in stratifying the stroke subtypes and severity and as a prognostic metric of secondary stroke occurrence (138, 139). Conversely, some have failed to replicate the association in their studies. Although those studies are presented with several limitations (140).

Eventually, extracellular vesicles (EVs) and their molecules are being investigated as biomarkers in stroke pathogenesis and in stratifying stroke subtypes (141). Platelet activation triggers the release of EVs. It is classified into three types based on their size and source: microvesicles, exosomes, and apoptotic bodies. It is regulated by the MISEV2018 guidelines recommended by “The International Society for Extracellular Vesicles (ISEV)” (142). Circulating EVs released from platelets stimulate endothelial cells and vascular smooth muscle cells, increasing vascular tissue inflammation and repair. The immunomodulatory role of platelet-derived EVs on CD4+ T cells in promoting platelet and fibrin aggregation and adhesion on vessel walls increases the risk of thrombus formation (143). Circulating EVs are elevated in patients with ACS and atherothrombotic incidents, especially in the initial hours of the event.

## Discussion

Currently, stroke management largely relies on empirical antiplatelet therapy, though many populations exhibit wide potential genetic variations leading to therapeutic failure, presenting with treatment complications and recurrent thrombotic events. Various genetic determinants of antiplatelet agents- aspirin, clopidogrel, prasugrel, and ticagrelor have been identified. They were studied to be associated with antiplatelet therapy efficacy, response, adverse events, and toxicity. Reduced response to antiplatelet therapy in patients with genetic variants has been studied aiding in therapy optimization. For example, patients with PlA1/A2 SNP of the GPIIIa receptor gene were demonstrated to have decreased response to aspirin (144). Likewise, drug toxicity in patients has been detected. For example, patients with CYP2C19 gain of function variants receiving clopidogrel therapy have a high risk of presenting with bleeding complications. Similarly, patients with rs5050 of angiotensinogen (AGT) gene receiving aspirin showed an elevated risk of peptic ulcer hemorrhage especially with genotype GG (145). The Clinical Pharmacogenetics Implementation Consortium (CPIC) tried to compile such adverse events related to genetic data in clinical algorithms for clopidogrel aiding in therapy optimization (146). This necessitates the detection of more genetic variants associated with antiplatelet drugs. With the advancement of high-throughput sequencing technologies, whole-genome sequencing in many populations has become possible. Newer genetic associations with clopidogrel response were detected by Genotype Information and Functional Testing (GIFT) exome study, ATP2B2, and TIAM2 through whole-exome sequencing (147). The number of physical, genetic, serum, and plasma biomarkers related to ischemic stroke has been identified. Specific miRNAs were found to be altered before the stroke occurrence, and these could be used as diagnostic and predictive biomarkers of stroke.

The clinical translation of pharmacogenomics testing in stroke management in using appropriate antiplatelet therapy will prevent adverse thrombotic events while improving therapeutic outcomes. Many studies have established the importance of platelet function testing (PFT)-guided antiplatelet therapy (148, 149). PFT is found to be more cost-effective in detecting antiplatelet response in comparison with genomic sequencing technologies (150). However, guidelines on PFT- or genotype-guided antiplatelet treatment are not well established given the ambiguity in studies (151, 152). A recent comparative study on PFTs on ischemic stroke patients has demonstrated that light transmittance aggregometry arachidonic acid platelet agonist (LTA-AA) and thromboelastographic arachidonic acid platelet agonist (TEG-AA) are effective in monitoring aspirin efficacy and response (78). Dual antiplatelet therapy (DAPT), comprising clopidogrel and aspirin is an effective strategy in managing the recurrence of stroke-related events. The dual-antiplatelet therapy (DAPT) score was developed to predict ischemic and bleeding risk in patients treated with percutaneous coronary intervention (PCI) (153, 154). The DAPT score and its decision tool was validated by several other studies including a meta-analysis, which concluded that it is helpful in characterizing ischaemic and bleeding events risk in post PCI patients and helps in deciding the desired duration of DAPT treatment (155). Another validated score in predicting bleeding complications while using DAPT is the PRECISE-DAPT score. The correlative analysis of genotypic data with clinical phenotyping data and platelet function tests will be a promising futuristic goal. This was achieved by Dewey et al. in their study, through whole-exome sequencing 50,000 subjects (88). Studies have been conducted, undertaking personalized approach based identified genetic variants. In stable CAD patients of the Chinese population, personalizing antiplatelet treatment based on maximum aggregation rate (MAR) in comparison with standard DAPT improved the health outcome after 180-day follow-up after PCI (156). According to a meta-analysis conducted recently in patients presenting with high platelet reactivity (HPR), platelet function test-based intensification of DAPT led to a reduction in adverse events (157). As diversity in both genotype and phenotype exists across different population groups, along with the need to determine the appropriate therapy for each individual, personalized medicine is the most promising futuristic approach in managing complex cerebrovascular events like acute ischemic stroke.

## Conclusions

The integration of specific biomarkers, genotype- as well as phenotype-related data in antiplatelet therapy stratification in patients with acute ischemic stroke will be of great clinical significance. However, the data on genetic determinants and biomarkers with specificity is limited. Ongoing and future clinical studies are hoped to yield further valuable evidence and standardized guidelines in translating a personalized approach to the management of ischemic stroke. This futuristic approach is believed to offer better management of thrombotic events while preventing stroke and antiplatelet drug-related complications.

## Author Contributions

PV, SP, and AA contributed to first draft of manuscript and acquisition of data. MM, MS, AA, and AK contributed to the analysis, interpretation, and critical revision of the manuscript for important intellectual content. MKS contributed to the literature review and critical revision. All authors contributed to the article and approved the submitted version.

## Conflict of Interest

The authors declare that the research was conducted in the absence of any commercial or financial relationships that could be construed as a potential conflict of interest.
